# *Cellulosimicrobium* sp. Strain L1: A Study on the Optimization of the Conditions and Performance of a Combined Biological Trickling Filter for Hydrogen Sulfide Degradation

**DOI:** 10.3390/microorganisms12081513

**Published:** 2024-07-24

**Authors:** Xuechun Wang, Xintian Li, Peng Hao, Xinran Duan, Yunhang Gao, Xiaojun Liang

**Affiliations:** 1 College of Veterinary Medicine, Jilin Agricultural University, Changchun 130118, China; 15243144321@163.com (X.W.);; 2 Institute of Animal Science, Ningxia Academy of Agriculture and Forestry, Yinchuan 750002, China

**Keywords:** *Cellulosimicrobium* sp. strain L1, biological trickling filter, hydrogen sulfide, thiosulfate, sulfate

## Abstract

Sulfide is a toxic and hazardous substance in the agricultural environment, which can cause damage to humans and livestock when exposed to large amounts of air. In this study, we performed one-factor optimization of the culture conditions and culture fractions of the *Cellulosimicrobium* sp. strain L1 and combined it with a biological trickling filter cell for the degradation of hydrogen sulfide for 24 consecutive days. The degradation effect of strain L1 and the biological trickling filter (BTF) on hydrogen sulfide was investigated, and the changes in intermediate products in the degradation process were briefly analyzed. The results showed that strain L1 had the highest conversion efficiency when incubated with 3 g/L sucrose as the carbon source and 1 g/L NH_4_Cl as the nitrogen source at a temperature of 35 °C, an initial pH of 5, and a NaCl concentration of 1%. The concentration of thiosulfate increased and then decreased during the degradation process, and the concentration of sulfate increased continuously. When strain L1 was applied to the biological trickling filter, it could degrade 359.53 mg/m^3^ of H_2_S. This study provides a deeper understanding of sulfide degradation in biological trickling filters and helps promote the development of desulfurization technology and the treatment of malodorous gasses produced by the accumulation of large quantities of livestock manure.

## 1. Introduction

Hydrogen sulfide is an irritant gas, and high concentrations of it are harmful to living organisms, while excessive inhalation may even lead to death [1]. In livestock and poultry farms, a large amount of accumulated livestock and poultry manure that is not cleaned up in a timely manner will produce malodorous gasses through fermentation, which are mainly dominated by hydrogen sulfide and ammonia [2,3]. Stirring and mixing can lead to the rapid release of hydrogen sulfide, increasing the risk of disease for animals and livestock workers [4,5]. Research has indicated that the concentration of hydrogen sulfide in livestock and poultry farms is related to the sulfate content in the water. In two pig houses, when using high-sulfate and low-sulfate well water on the same farm, the daily average hydrogen sulfide concentration detected within two years differs by about 5.6 times. Currently, with the rapid development of the farming industry, more than 90 million cattle and 150 million pigs are kept on farms [6], generating about 3.8 billion tons of animal manure annually [7]. While this leads to a pressing need for manure disposal, the concentration of hydrogen sulfide in farms has also increased. There have been several reported incidents of the poisoning of workers and the death of animals caused by high concentrations of hydrogen sulfide in poultry farms [8,9]. Hydrogen sulfide in farms can cause damage to the animal organism, with low concentrations causing inflammation, and when the concentration of hydrogen sulfide reaches 50–200 ppm, it can lead to symptoms such as vomiting and diarrhea in pigs [10]. Inhaling hydrogen sulfide can have more serious effects on humans: the sense of smell is lost when hydrogen sulfide concentrations are between 100 and 250 ppm. At concentrations of 250 to 500 ppm, hydrogen sulfide causes excitation, headaches, cyanosis, and pulmonary edema. Concentrations above 500 ppm cause nausea, dizziness, and shortness of breath, which can lead to unconsciousness, apnea, and even death after more than 30 min of exposure [11]. Therefore, the treatment of hydrogen sulfide in livestock farms is crucial. It has been shown that the concentration of hydrogen sulfide in livestock farms is related to the sulfate content of the water, with an approximate 5.6-fold difference in the average daily hydrogen sulfide concentration detected over a two-year period in two piggeries on the same farm using high-sulfate and low-sulfate well water [12].

To date, the methods for removing hydrogen sulfide are broadly classified into two categories: physicochemical and biological technologies, which can be further divided into wet absorption, adsorption, membrane separation, metal oxide catalysis, and biological treatment [13]. Wet absorption is the main technology for removing hydrogen sulfide; however, the most representative alkanolamine absorption method has the disadvantages of high regeneration energy consumption, a corrosive absorption process, large reagent loss, and it easily causes secondary pollution [14]. Adsorption is a commonly used method for removing hydrogen sulfide from water, but there are also problems with this method, such as a low adsorption capacity and a long contact time, and many adsorbent surfaces are irreversible chemical reactions that will result in a high loss. The membrane separation method has good adaptability but poor corrosion resistance, resulting in a short service life and high application costs [15], while the metal oxide catalytic method mainly uses a variety of common metal-based catalysts to remove hydrogen sulfide. The main drawbacks of this technology are that the catalyst is easy to deactivate, there are regeneration difficulties, and it has a high cost [16], while in contrast, biological treatment has the advantages of a low cost, high efficiency, and the fact that it does not cause secondary pollution, and it has been proven to be very suitable for the removal of hydrogen sulfide [17]. Among them, biological trickling filter (BTF) systems are easy to control and have low operating costs and a high removal efficiency.

In recent years, biological trickling filters (BTFs) have been widely used for the treatment of volatile organic compounds (VOCs) and malodorous compounds [18]. Under anaerobic conditions, BTFs can use nitrate and nitrogen dioxide as electron acceptors to remove hydrogen sulfide [19], but the process is complex and difficult to control. In contrast, aerobic BTFs can utilize oxygen from the air as an electron acceptor [20]. Some studies have shown that aerobic BTFs can effectively remove high concentrations of hydrogen sulfide [21]. In addition, it has been shown that the use of pure monoculture bacteria in the biological trickling filter process is more advantageous than the use of mixed strains, as it has a shorter start-up time, is simpler to operate, and results in a higher H_2_S removal efficiency [22]. Moreover, one study compared the use of aerobic and anaerobic biological trickling filter tanks for degrading volatile compounds, and the results showed that aerobic BTFs have higher degradation rates [21].

In this study, strain L1 was inoculated in reticulated polyurethane foam used as a BTF filler for BTF degradation of H_2_S, a typical malodorous gas released from farm cesspool treatments, to investigate the effect of the H_2_S concentration on BTF degradation. In addition, the immobilization effect of *Cellulosimicrobium* sp L1 and the removal rate of hydrogen sulfide in the biological trickling filter (BTF) system were evaluated. This experiment was conducted with the aim of providing an advantageous resource for the biological removal of hydrogen sulfide and a reference for the removal of H_2_S by means of pure culture bacteria combined with a BTF.

## 2. Materials and Methods

### 2.1. Materials

*Cellulosimicrobium* sp. L1 is a laboratory-conserved strain of a functional bacterium with the ability to degrade hydrogen sulfide (GeneBank:MZ687074) [23]. The biological drip filter was purchased from Shanghai Binzhi Organic Glass (Shanghai, China); four LZB-3WBF gas flow meters (6–60 mL/min, 30–300 mL/min, 16–160 L/h, 0.15–1.5 L/min) and an LZB-4WBF liquid flow meter (25–250 mL/min) were purchased from the Shengjie flagship store; the Diaphragm pressure gauge was purchased from Shanghai Lianli Instrument (Shanghai, China); the portable pump suction hydrogen sulfide detector was purchased from Zhonggong Tiandi Technology (Beijing, China), and the air pump was purchased from Suzhou Litehai Boosting Technology (Suzhou, China).

### 2.2. Growth Characteristics and Scanning Electron Microscope (SEM) Observation of Strain L1

Strain L1 was inoculated into TSB at 1% (*v*/*v*), sampled at fixed time intervals, and assayed for OD_600_ nm (UV/visible spectrophotometer MU701, Shimadzu, Kyoto, Japan). Sterilized TSB was used as a control, and the growth curve was determined by plotting the values at each time point. The bacterial cultures were centrifuged (3000 revolutions per minute (rpm), 10 min, 4 °C), the supernatant was discarded, and 2.5% glutaraldehyde solution (electron microscope fixative) was added and fixed for 12 h at 4 °C. The bacteria were collected and washed three times with PBS solution and then centrifuged again. Thereafter, the bacteria were sequentially exposed to 10%, 30%, 50%, 70%, and 90% ethanol for 5 min each for dehydration. Finally, the treated samples were subjected to scanning electron microscopy (SEM, Shanghai GECHUANG TEST Technology Center, Shanghai, China).

### 2.3. Optimization of Degradation Conditions

#### 2.3.1. Single Factor Test

In order to obtain a good degradation effect, the medium’s composition and culture conditions were optimized. First, the effects of different substrate concentrations (0.1, 0.3, 0.5, 0.7, and 1 g/L) on the growth and sulfur conversion of the strains were explored. The culture fractions were optimized under laboratory conditions using various carbon sources (sucrose, glucose, CH_3_COONa (Kaiyuan Chemical Reagent, Kaiyuan, China), sodium citrate, mannitol) at 0.5 g/L and nitrogen sources (NH_4_Cl, NH_4_H_2_PO_4_, ammonium tartrate, NaNO_3_, urea, and yeast powder) at 1 g/L. To obtain the optimal culture conditions, strain L1 was cultured at different temperatures (20 °C, 25 °C, 30 °C, 35 °C, 40 °C), different initial pH values (3, 5, 7, 9, 11), and different NaCl concentrations (0.1%, 0.5%, 1.0%, 1.5%, 2.0%).

#### 2.3.2. Response Surface Optimization Experiment

By combining the results of the one-factor experiments, a three-factor, three-level response surface experimental program was designed for the culture temperature, initial pH, and NaCl concentration of the strains using the Box–Behnken response surface method. The response surface analysis was performed using Design Expert 8.0 software, and the experimental factor levels and codes are shown in Table 1.

### 2.4. Experimental Setup

**Figure 1:**
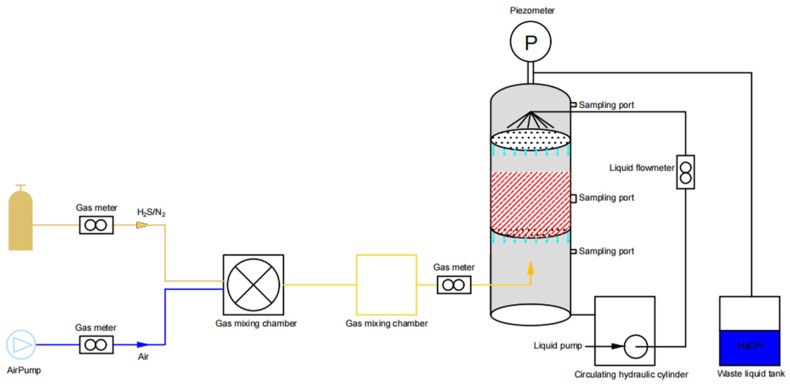
Schematic diagram of biological trickling filter.

The BTF system consisted of a biological trickling filter tower, an air intake system, and a nutrient recirculation system (Figure 1). The BTF tower was composed of Plexiglas with an inner diameter of 200 mm and a height of 0.8 m. The system was designed with a high density of 0.8 m and an inner diameter of 200 mm. Reticulated polyurethane foam cubes were used as packing material with an effective height of 0.2–0.25 m. In the air intake system, hydrogen sulfide was transported to the mixing chamber through entrained air. The operation was a gas–liquid countercurrent, and the gas passed through the biological trickling filter (BTF) from bottom to top. The bottom of the BTF was connected to a water tank, and the circulating nutrient solution in the tank was uniformly injected into the reactor from the top by a peristaltic pump. The circulating nutrient solution not only provided a certain amount of nutrient solution for the microorganisms attached to the packing material but also ensured sufficient humidity inside the reactor, providing the basic conditions for gas–liquid mass transfer.

### 2.5. Operating Conditions

The entire test of the BTF was carried out over 24 days. The operating time was divided into four phases based on the empty bed residence time (EBRT) (Table 2). At the end of each cycle, 650 mL of fresh recirculating fluid was used instead of 650 mL of recirculating fluid in the tank. During the operation, 60 mL of solution from the recirculating fluid tank was used for analysis and was replaced with 60 mL of fresh recirculating fluid each day. The pH of the recirculating liquid was tested periodically. Prior to the experiments, the molar volume of the gas was assumed to be 22.4 mol/L, and the concentration of H_2_S in the inlet gas was kept at 5000 ppmv. Figure 1 shows the process of H_2_S degradation in the BTF: the gas mixture was passed through the gas flowmeter using a gas–liquid countercurrent operation and was formulated to have different H_2_S concentrations. It then entered the BTF from the bottom, passed through the filler layer of the packing material, and was located at the top of the reactor. Finally, the gas passed through a saturated NaOH solution. The bottom of the BTF was connected to a water tank, in which the recirculating nutrient solution was uniformly injected into the reactor from the top by a peristaltic pump. The recirculating nutrient solution not only provided some nutrients for the microorganisms attached to the packing but also ensured sufficient humidity inside the reactor, thus providing the basic conditions for gas–liquid mass transfer. The volume of the circulating nutrient solution tank was 12 L. The formula of the circulating solution is shown in Table 2. During the experimental period, half of the circulating fluid was replaced every 5 days. The H_2_S removal efficiency (RE) was affected by several factors such as the loading rate (LR), the H_2_S concentration, the removal capacity (EC), the EBRT, the packing height, and the gas–liquid ratio [24]. These parameters were calculated using Equations (1)–(4). The H_2_S inlet and outlet concentrations were detected using a composite gas detector portable instrument (H_2_S-800, Zhonggong Tiandi Technology China, Beijing, China) based on the electrochemical detection principle.

RE = (*C_in_ − C_out_*)/*C_in_* × 100% (1)

LR = (*Q* × *C_in_*)/V (2)

EC = *Q*/V × (*C_in_* − *C_out_*) (3)

EBRT = V/*Q* (4)

### 2.6. Sampling and Analysis

The gas flow rate was measured using rotameters (VA, Dwyer, Michigan City, IN, USA). After sampling and diluting the gas from the inlet and outlet gasses, the H_2_S concentration of the inlet and output gasses was determined using a hydrogen sulfide analyzer (GasAlert Micro 5, BW technology from Honeywell, Mississauga, ON, Canada). The measurement range was 0–500 ppmv and the sensitivity was 1.0 ppmv. The pH of the recirculated liquid in the recirculating liquid tank was measured using a pH meter (CLEAN INSRUMENTS CO, Shanghai, China). Biofilm samples were collected from the packaging material on days 0, 16, and 24 and immediately transferred into sterile Eppendorf tubes.

The inlet and outlet concentrations of H_2_S were measured using a gas chromatography instrument (Techcomp GC7900, Hong Kong, China). The content of S^2−^ was determined by means of *p*-amino-dimethylaniline spectrophotometry [25].

## 3. Results and Discussion

### 3.1. Growth Characteristics and SEM Observation Results of Strain L1

The growth curve of strain L1 is shown in Figure S1. Strain L1 showed logarithmic growth at 6–14 h, after which the growth rate of the strain entered a slow stage and a stable growth period. The morphological characterization of strain L1 showed that it is in accordance with the findings of Li [23].

### 3.2. Effects of Different Conditions on Sulfur Conversion Efficiency

#### 3.2.1. The Effect of Different Substrate Concentrations on Bacterial Growth and Sulfur Conversion

**Figure 2:**
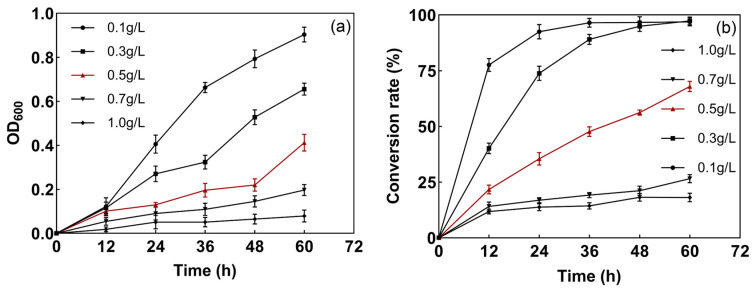
Effect of S^2−^ concentration on bacterial growth (**a**) and degradation efficiency (**b**).

The degradation experiments employed a mineral salt media (MSM, (KH_2_PO_4_, MgCl_2_·6H_2_O, K_2_HPO_4_, NaHCO_3_, NH_4_Cl, NaCl, sucrose)). The growth rates of the strains were different when the substrate concentrations were different, as shown in Figure 2. When the substrate concentration was lower than 0.5 g/L, strain L1 grew well and transformed S^2−^ efficiently, whereas, when the S^2−^ concentration was 1 g/L, the growth of the strains was slow and the transformation rate decreased. This may be because the high concentration of S^2−^ increased the osmotic pressure of the medium, which inhibited the transporter system of the strain and decreased its S^2−^ conversion ability. In addition, the accumulation of sulfate during the transformation process may have caused biotoxicity for the strain, inhibiting its growth and reducing the degradation of S^2−^.

#### 3.2.2. The Influence of Different Carbon and Nitrogen Sources and Their Concentrations on the Sulfur Conversion Efficiency

**Figure 3:**
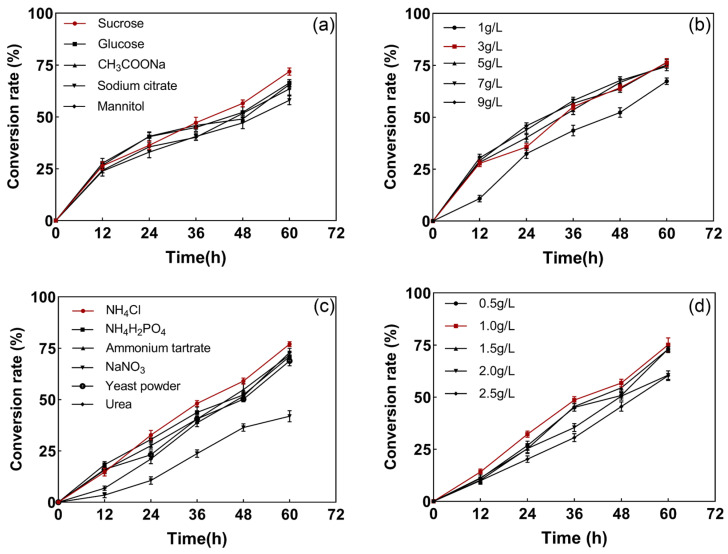
Degradation efficiency of S^2−^ under different carbon sources and carbon source concentrations (**a**,**b**) and different nitrogen sources and nitrogen source concentrations (**c**,**d**).

The ability of the strains to vary in S^2−^ their conversion efficiency under different carbon and nitrogen sources was different, as shown in Figure 3a. Sucrose showed the highest conversion efficiency of 75%, which may have been due to the fact that sucrose as a carbon source is usually considered to be more favorable to bacterial respiratory metabolism than glucose and fructose alone, which leads to more energy for proliferation and differentiation. Moreover, sucrose is inexpensive and stable, making it suitable for the mass culture of bacterial strains. For the carbon source, as the first substrate of cellular metabolism, fewer catabolic steps are more suitable for substrate growth evolution [26]. As shown in Figure 3b, the higher the concentration of sucrose, the higher the strain’s conversion rate of sulfur, indicating that the strain grew well and the growth rate accelerated with an increase in the sucrose concentration. However, after a certain concentration, a relative saturation state was reached, and the results of the conversion rate did not differ much, so a sucrose concentration of 1.0 g/L was selected for economic considerations. As shown in Figure 3c, under different nitrogen source conditions, the strain was most efficiently transformed in the medium supplemented with NH_4_Cl, converting 75% of S^2−^ at 60 h. The optimum conversion efficiency of the strain was achieved at an NH_4_Cl concentration of 1.0 g/L (Figure 3d). It has been shown that different nitrogen sources and inoculum levels have a non-negligible effect on biological growth [27]. Aliabadi, Chen, and Lu et al. similarly found that bacteria can proliferate organic and inorganic nitrogen during sulfur conversion and may have multiple nitrogen metabolism pathways [28,29,30]. In Xu’s study, it was likewise found that his strain was more suitable for a low carbon-to-nitrogen ratio in the growth environment [31]. Multiple metabolic pathways are more suitable for practical applications in wastewater environments with complex conditions.

#### 3.2.3. Effects of Different Salt Concentrations, Temperatures, and Initial pH Values on Sulfur Degradation Efficiency

**Figure 4:**
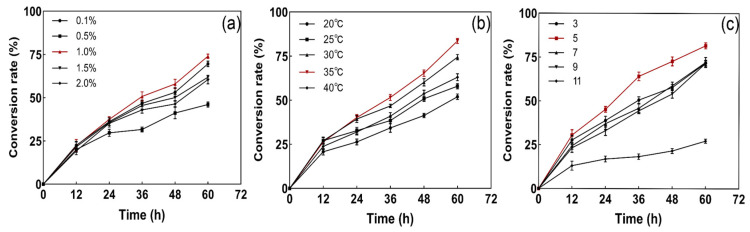
The strain’s degradation efficiency of S^2−^ under different NaCl concentrations (**a**), temperatures (**b**), and initial pH values (**c**).

In this study, it was found that the strain converted sulfide best at a 1% NaCl concentration (Figure 4a). Therefore, to improve the sulfur conversion, we chose a 1% NaCl concentration. The effect of the incubation temperature on the sulfide conversion of the strain was determined by fermentation at different temperatures ranging from 20 to 40 °C (Figure 4b). The results showed that the strain could achieve maximum sulfide conversion at 35 °C.

The efficiency of the strain’s S^2−^ conversion was different under different initial pH conditions. From Figure 4c, it can be seen that the conversion efficiency of S^2−^ was highest at an initial pH of 5, and its corresponding degradation rate reached 80%. The chemical and biological oxidation of sulfide occur simultaneously, and different temperatures, pH values, and ionic strengths affect the rate of chemical oxidation [31]. pH plays an important role in the production of microbial enzymes [29]. Hydrogen sulfide is an acidic gas that is soluble in water, and its chemical reaction in alkaline solution results in the formation of sulfide and water, hence the initial reaction has an acidic pH. The metabolic efficiency is extremely high when the microorganisms are at the optimal pH in terms of the effects on their biological activity [32].

#### 3.2.4. Response Surface Experiment

**Figure 5:**
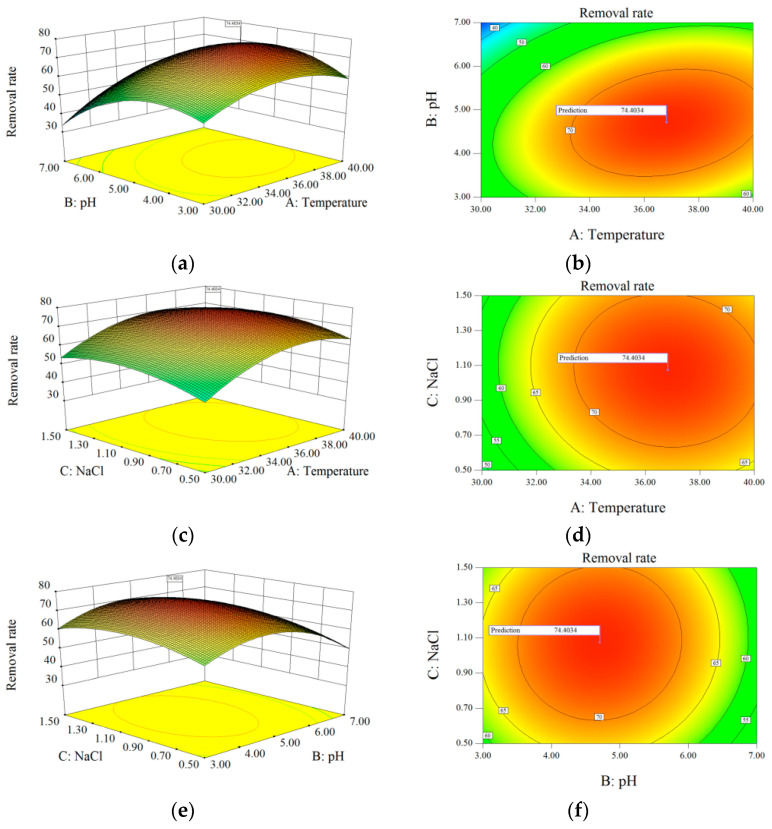
Three-dimensional and two-dimensional response surface plots for optimizing S^2−^ removal using (**a**,**b**) pH and temperature; (**c**,**d**) temperature and NaCl; and (**e**,**f**) NaCl and pH as the interactive parameters.

In order to improve the strain’s S^2−^ degradation efficiency, this paper also used a three-factor, three-energy response surface analysis test to optimize the conditions of the strain based on a one-factor experiment [26]. This was based on the Box–Behnken central composite design principle, with the temperature (a), initial pH (B), and NaCl concentration (C) as the three independent variables (Table 1) and the sulfide conversion rate as the dependent variable, and the results are shown in Figure 5.

Multiple regression fitting of the data in Table S1 resulted in a binary multiple regression equation for the S^2−^ conversion rate based on the temperature, initial pH, and NaCl concentration: S^2−^ conversion rate = +72.49+7.55×A−5.42×B+1.97×C+4.70×A×B−0.63×A×C $+\;0.65\times B\times C-9.29\times A^{2}-12.25\times B^{2}-5.69\times C^{2}$. The model selected in Table S2 (*p* < 0.01) indicates that the developed model is correct and that the equation is well-fitted. The determined model correction coefficient R^2^ was 0.9203, indicating that the model explained 92.03% of the variation in the response values and that the model fitted the data well. Therefore, the model is reasonable and effective at optimizing the conditions for the transformation of sulfide by the strain.

### 3.3. Changes in Individual Metabolites during S^2−^ Transformation Process

**Figure 6:**
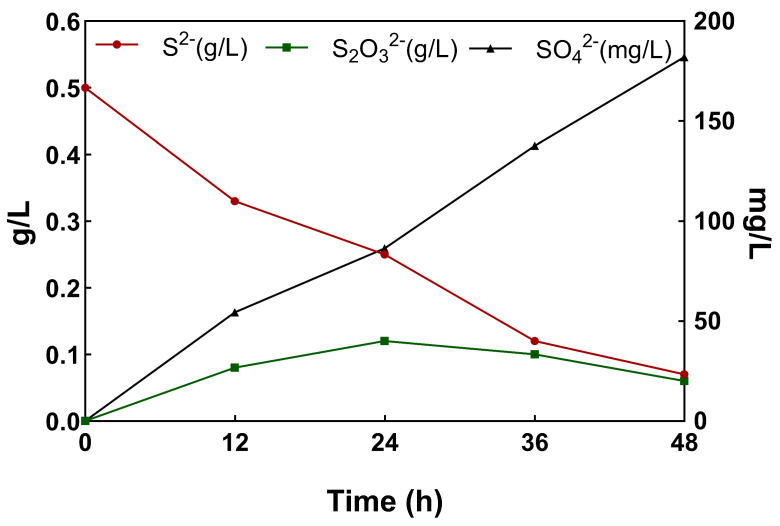
Dynamic changes in various metabolites during the BTF of S^2−^.

The strain was cultivated under optimal conditions, and the changes in the concentrations of S^2−^, S_2_O_3_^2−^, and SO_4_^2−^ in the solution are shown in Figure 6. According to the results of this paper, it can be inferred that S^2−^ was rapidly oxidized to generate S_2_O_3_^2−^ in the solution at the early stage of conversion and then further oxidized to generate SO_4_^2−^. The conversion rate of the strain could reach more than 86% when cultured for 48 h, with superior sulfur conversion performance. The oxidation of sulfide to sulfate and thiosulfate is an indispensable core step in the sulfur oxidation process [33], and it is speculated that S_2_O_3_^2−^ production may be due to the different pathways of sulfide conversion under oxygen-limited conditions [34], while the continuous production of sulfate is due to the presence of a periplasmic enzyme system in the bacteria, which can oxidize sulfur and sulfide to sulfate [35]. It has also been demonstrated that E. coli can convert thiosulfate to thioalkyl sulfide [36].

### 3.4. Application of Biological Trickling Filter

**Figure 7:**
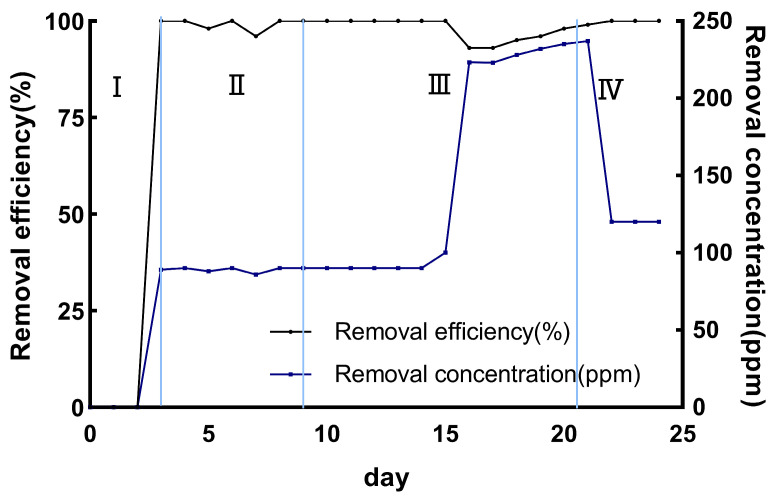
H_2_S removal concentration and removal efficiency in BTF. (I, II, III, IV in the figure represent the four stages, and the detailed conditions are explained in Table 2).

In this experiment, the gas–liquid reverse circulation hanging film initiation method was used to construct the biological trickling filter system, and the biological trickling filter system worked continuously for 25 days, while the H_2_S concentration in the inlet and outlet of the drip filter tower was measured every day. Prior to the strain mounting, a certain concentration of hydrogen sulfide was passed into the biological trickling filter, and the inlet and outlet concentrations were measured. It was found that there was no difference in the concentration, thus indicating that the packed polyurethane foam had no adsorption function for hydrogen sulfide, there was no degradation, and the formal experiments could be started. As shown in Figure 7, the stage I EBRT was 8 min, which is the strain hanging phase. The stage Ⅱ EBRT was 8 min, and the removal rate of H_2_S reached 99 ± 1% when the aeration concentration was 90 ppm. The stage Ⅲ EBRT was 5 min, and the removal rate reached 100% when the aeration concentration was 90 ppm, while the removal rate was 96 ± 3% when the aeration concentration was 240 ppm. The stage Ⅳ EBRT was 2 min, and the removal rate was 100% when the aeration concentration was 120 ppm. Many studies have shown that strain-associated bio-drip filters have the function of efficiently removing harmful gasses, and their degradation effect is higher than that of the biodegradation method using degrading bacteria alone. Previously, 2-chlorotoluene and acetone were removed using a combined biofiltration tank with degrading strains [37,38]. In a study by Marycz M, the ability of conventional biofilters and biological trickling filters to degrade VOCs was compared [39,40]. We chose reticulated polyurethane foam as the packing material for the biological trickling filter cell, which has the advantage of being easily adsorbed and loose and porous, and it can create the bacterial strains’ hanging films more easily. Polyurethane foam was also chosen as a filler for biodrip filters to remove hazardous substances in a study by Bonilla-Blancas W, Dobslaw D, et al. [38,41].

The changes in the concentration of each ion in the circulating liquid cylinder during the operation of the biological trickling filter tank were determined, and the results are shown in Figure S2. It can be seen that after the initial addition of S^2−^ was transformed, no more was generated, while with the passage of hydrogen sulfide, the S_2_O_3_^2−^ in the solution increased first, and at the late stage of aeration, S_2_O_3_^2−^ increased and then decreased, while SO_4_^2−^ increased throughout. So, it was judged that SO_4_^2−^ was the end product of the oxidation of S^2−^. The reason for the constant increase in SO_4_^2−^ was the accumulation of sulfur monomers (S0) in biological systems due to the oxidation of sulfur monomers [42], and S_2_O_3_^2−^ also contributes to the generation of SO_4_^2−^ by initiating free radical chain reactions [43]. In addition, during the reaction in the biological trickling filter cell, the sulfur oxidation pathway is different for different levels of oxygen content and, therefore, its products are different [44]. In a study of denitrification using sulfide as an electron donor, S^2−^ and S_2_O_3_^2−^ were consumed as a sulfur source, provided by the denitrification process, and the amount of sulfate accumulated and the concentration increased throughout the denitrification process, confirming that sulfate was the final product of the sulfur conversion process [45].

### 3.5. Changes in Fillers in BTF

**Figure 8:**
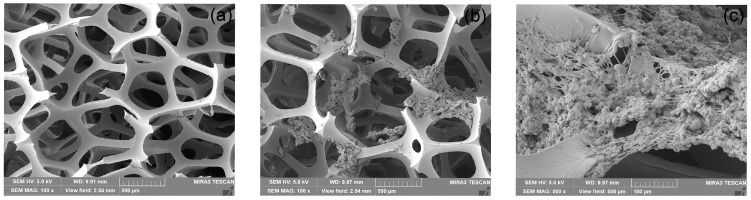
Scanning electron microscope observation of the immobilization of strains. ((**a**) shows the scanning electron microscope image of the packing at 100× before the strain was mounted, (**b**,**c**) show the scanning electron microscope image of the packing at 100 and 500× on day 24 when the strain was mounted).

The hang-up of strain L1 in the polyurethane foam was determined by comparing the scanning electron micrographs of the fillers on day 0 and day 24 of the experimental run, and Figure 8 presents scanning electron microscopy (SEM) images of the reticulated polyurethane foam filler and the microorganism-inoculated filler on day 24. As shown in Figure 8a, the initial surface of the reticulated polyurethane foam cube filler was smooth in the absence of microbial inoculation. Figure 8b and 8c show the overall and localized electron micrographs of the polyurethane foam packing during the operation of the BTF unit, and the comparison shows the presence of microorganisms on the surface of the packing, which indicates that the bacterial strains completed the film hanging in the polyurethane foam and were well immobilized. Twenty-four days after the introduction of hydrogen sulfide, obvious could be observed on the filler’s surface: the original filler structure was destroyed and many short rod-shaped microorganisms were attached. The strains attached to the filler thus achieved degradation [46].

## 4. Conclusions

In this study, we optimized the culture composition and conditions for S^2−^ removal using strain L1 and found that strain L1 has the ability to degrade S^2−^ efficiently. When the S^2−^ conversion performance of strain L1 was measured, it was found that the final product of S^2−^ conversion was SO_4_^2−^. When strain L1 was applied to biological trickling filtration, the strain completed the membrane hanging in the filler, which indicated that the BTF had the ability to degrade H_2_S efficiently in the stable operation stage and provided theoretical support for the biodegradation of H_2_S.

## Figures and Tables

**Table 1:** Factor level and coding of the removal rate of optimizing strain L1 S^2−^ by means of the response surface methodology.

Level	A-Temperature (°C)	B-Initial pH	C-NaCl Concentration
−1	30	3	0.5%
0	35	5	1%
1	40	7	1.5%

**Table 2:** Operating conditions of BTF.

Stage	Time (d)	EBRT	Gas Flow Rate (mL/min)	Inlet Concentration (ppm)	Outlet Concentration (ppm)	Maximum Removal Capacity (g/(m^3^·h))	Removal Rate (%)	Temperature (°C)
I	0–3	8 min	360	0	0	0	0	
II	4–9	8 min	360	90	1 ± 1	939.19	99 ± 1.53%	15
III	10–15	5 min	600	90	0	1565.31	100%	16 ± 1
	16–21	5 min	600	240	9 ± 5.02	4174.17	96.75 ± 3.25%	12 ± 2
IV	22–24	2 min	1500	120	0	5217.71	100%	13 ± 2

## Data Availability

The data will be made available upon request.

## Supplementary Materials

The following supporting information can be downloaded at https://www.mdpi.com/article/10.3390/microorganisms12081513/s1, Figure S1: The result of bacteria growth curves; Figure S2: Change of ion concentration in circulating fluid cylinder; Table S1: Response surface method design test scheme and results; Table S2: Model regression analysis.
