# Supplementary material for: Cellulosimicrobium sp. Strain L1: A Study on the Optimization of the Conditions and Performance of a Combined Biological Trickling Filter for Hydrogen Sulfide Degradation
Source: Microorganisms. 2024 Jul 24;12(8):1513. doi: 10.3390/microorganisms12081513 (PMC11356333; doi:10.3390/microorganisms12081513)
Supplement: Supplementary file 1 [file microorganisms-12-01513-s001.zip › microorganisms-3042697-supplementary.pdf]

Cellulosimicrobium sp. Strain L1: A study on the Optimization of the Conditions and Performance of a Combined Biological Trickling Filter for Hydrogen Sulfide Degradation

Xuechun Wang[1], Xintian Li[1], Peng Hao[1], Xinran Duan[1], Yunhang Gao[1] *, Xiaojun Liang[2]

[1] College of Veterinary Medicine, Jilin Agricultural University, Changchun 130118, China.

*Corresponding authors: Tel.: +86-13159752912

Email: gaoyunhang@163.com (Yunhang Gao).

[2] Institute of Animal Science, Ningxia Academy of Agriculture and Forestry, Yinchuan, 750002, China

lxj0520@163.com

Total number of pages: 4;
Total number of Figures: 2
Total number of Tables: 2

Figure Captions

Fig. S1. The result of bacteria growth curves.

Fig. S2. Change of ion concentration in circulating fluid cylinder.

Table Captions

Table S1. Response surface method design test scheme and results.

Table S2. Model regression analysis.

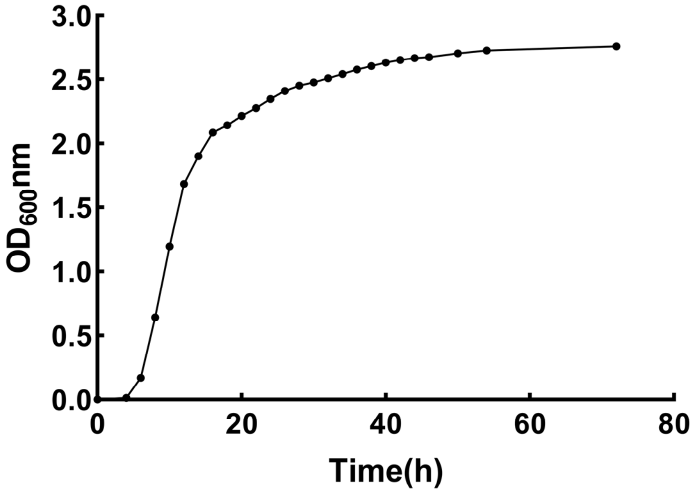

Fig. S1. The result of bacteria growth curves.

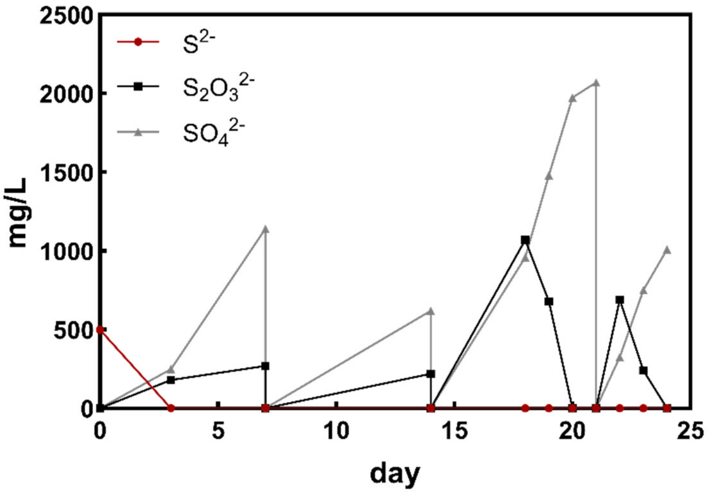

Fig. S2. Change of ion concentration in circulating fluid cylinder.

Table S1. Response surface method design test scheme and results.

Group	Temperature	pH	NaCl concentration	S^{2-} Removal rate (%)
1	35	5	1	73.16
2	35	3	1.5	66.64
3	30	5	1.5	53.90
4	40	3	1	48.91
5	35	3	0.5	57.31
6	35	5	1	77.27
7	40	7	1	37.48
8	30	3	1	52.82
9	30	5	0.5	50.14
10	40	5	1.5	50.34
11	40	5	0.5	34.39
12	35	5	1	75.27
13	30	7	1	39.60
14	35	7	0.5	45.14
15	35	5	1	76.92
16	35	7	1.5	57.10
17	35	5	1	76.62

Table S2. Model regression analysis.

Source	SS	d*f*	MS	*F*-Value	*P*-Value	
Model	2062.68	9	229.19	8.99	0.0042	**
A- T	455.87	1	455.87	17.88	0.0039	**
B- pH	234.69	1	234.69	9.21	0.0190	*
C-NaCl	30.97	1	30.97	1.21	0.3068	NS
AB	88.27	1	88.27	3.46	0.1051	NS
AC	1.61	1	1.61	0.063	0.8086	NS
BC	1.72	1	1.72	0.067	0.8028	NS
A^2	363.21	1	363.21	14.25	0.0069	**
B^2	632.13	1	632.13	24.79	0.0016	**
C^2	136.33	1	136.33	5.35	0.0540	NS
Residual	178.46	7	25.49	-	-	
Lack of Fit	148.23	3	49.41	6.54	0.0507	NS
Pure Error	30.23	4	7.56	-	-	-
Cor Total	2241.14	16	-	-	-	-

SS：square sum； d*f*：degrees of freedom；MS：mean square ***：Glaring（*P*-Value <0.001）；**：Remarkable（*P*-Value < 0.01）; *：Statistically significant （*P*-Value < 0.05）; NS：Not significant （*P*-Value > 0.05）
